# Neural correlates of fatigue after traumatic brain injury

**DOI:** 10.1093/braincomms/fcaf082

**Published:** 2025-02-26

**Authors:** Annina E Anliker, Léa A S Chauvigné, Leslie Allaman, Adrian G Guggisberg

**Affiliations:** School of Health Professions, Physiotherapy, Bern University of Applied Sciences, Berne 3007, Switzerland; Division of Neurorehabilitation, Department of Clinical Neurosciences, University Hospitals of Geneva, Geneva 14 1211, Switzerland; Division of Neurorehabilitation, Department of Clinical Neurosciences, University Hospitals of Geneva, Geneva 14 1211, Switzerland; Division of Neurorehabilitation, Department of Clinical Neurosciences, University Hospitals of Geneva, Geneva 14 1211, Switzerland; Division of Neurorehabilitation, Department of Neurology, University Hospital of Bern, Inselspital, Berne 3010, Switzerland

**Keywords:** EEG, functional connectivity, two-back task, multivariate prediction, network

## Abstract

Fatigue is the main cause of disability after traumatic brain injury and has negative impact on social, physical and cognitive functions, participation in daily activities, and ability to work. Since the neural underpinnings are largely unknown, few causal treatments are currently available. This study therefore aimed to investigate the neural correlates of subjective fatigue after traumatic brain injury, controlling for differences in cognitive performance, motor performance and subjective psychological covariates such as depression, anxiety and apathy. Seventeen chronic traumatic brain injury patients (10 with and seven without fatigue) and 11 age, sex, and education-matched healthy controls participated in the study. The dependent variable, overall fatigue, was quantified as the sum of the subscales of the multivariate fatigue inventory. Subjective psychological covariates were extracted from appropriate questionnaires. Brain activation during a two-back task and functional connectivity at rest were reconstructed from high-density EEG. Cortical excitability was quantified from motor evoked potentials induced by transcranial magnetic stimulation over the primary motor cortex. Cognitive performance was assessed with a two-back task as well as with a comprehensive neuropsychological test battery. Motor performance was quantified with Jamar dynamometer. Beside the between-group differences in most fatigue subscales resulting from the group attribution, participants also differed in subjective memory functions, depression, anxiety and apathy. Conversely, objective neuropsychological performance was similar across groups in most domains, except for alertness and divided attention (*P* ≤ 0.039). At the neural level, we observed no difference in corticospinal excitability, but a significant disruption of global resting-state alpha-band functional connectivity between cortical midline structures and the rest of the brain in patients with fatigue (*P* = 0.006). Furthermore, individuals with fatigue exhibited reduced signs of overall brain activation compared with healthy controls throughout the cognitive task (*P* = 0.032) without time-on-task effect. In a multivariate regression model, resting-state functional connectivity (*P* = 0.013) and subjective psychological questionnaire scores (*P* < 0.0001) were independent predictors of fatigue. In conclusion, our results suggest that disrupted network interactions are the primary independent neural predictor of fatigue. This may serve as a new target for therapy.

## Teaser

Fatigue is a major cause of disability after traumatic brain injury. Anliker *et al*. studied the neural correlates using EEG, finding disrupted resting-state alpha-band connectivity and reduced brain activation during tasks in fatigued patients. These findings may suggest new therapeutic targets.

## Graphical Abstract

**Graphical Abstract:**
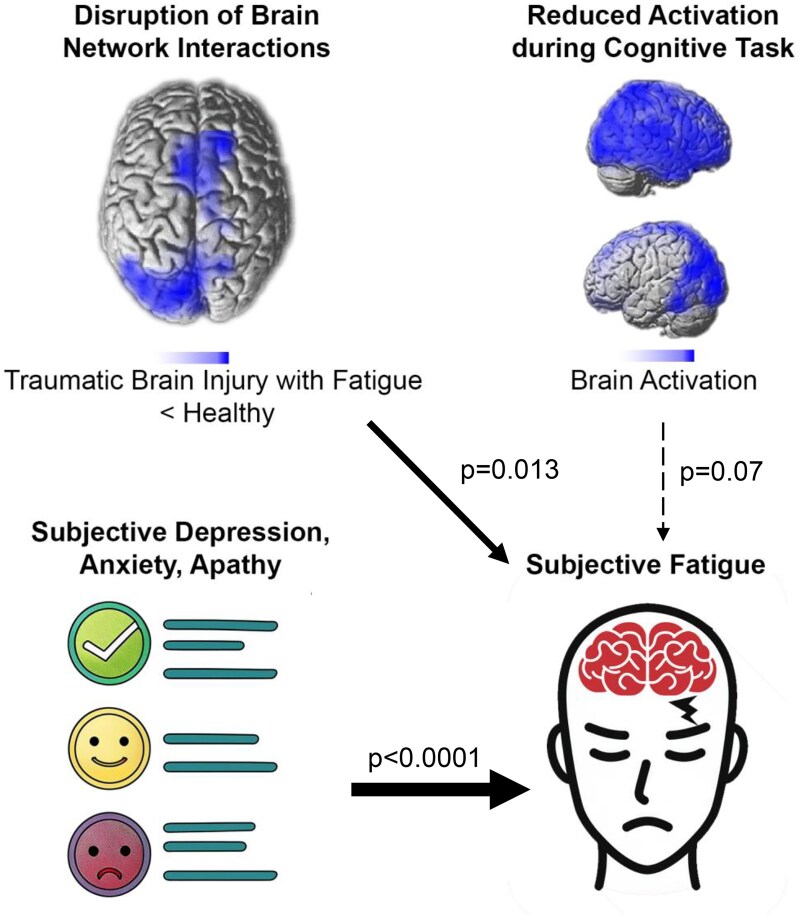

## Introduction

Fatigue could be defined as difficulty in initiating or sustaining voluntary activities.^1^ Physiological fatigue acts as a protective function to avoid harmful consequences of excessive effort (e.g. decreased muscular strength).^2^ It can occur on mental and physical levels.^3^ On the other hand, pathological fatigue is not related to the exertion level of a task, is exaggerated and needs more recovery time.^2^ Fatigue is a common symptom after traumatic brain injury (TBI). It is present in 21–73% of patients,^4^ and it does not seem to be significantly dependent on the severity nor the time since the injury.^2^ Palm *et al*.^3^ found fatigue to be the main cause of disability after TBI. It has a negative effect on social, physical and cognitive functions^5^ as well as participation in daily activities and social life and therefore quality of life.^3,6^ Furthermore, Palm *et al*.^3^ found a correlation between higher level of mental fatigue and lower employment status.

Despite the high burden, little is known about the specific clinical, behavioural and physiological mechanisms of fatigue after TBI.^4^ On the psychological level, Chaudhuri and Behan^1^ suggest that fatigue could occur because of a loss of interest and motivation and the lack of feedback from motor, sensory and cognitive systems that indicate the level of perceived exertion. Furthermore, Schönberger *et al*.^7^ found fatigue to be predictive of depression and sleepiness. Findings suggest that fatigue after TBI or post-stroke is a cause, not a consequence, of anxiety, depression and daytime sleepiness.^8-10^

On the behavioural level, Dillon *et al*.^11^ describe relatively weak evidence for a relationship between cognitive impairment like information processing, attention, memory and executive function and higher fatigue levels after acquired brain injury. Azouvi *et al*.^12^ found that TBI patients reported significantly higher subjective mental effort while completing a divided attention task, although they were just as able as healthy controls to perform the task.

The neural mechanisms underlying pathological fatigue in patients with acquired brain lesions such as TBI have received surprisingly little attention. Lesion studies have often been unable to identify associations with damage to specific brain regions^13,14^ or have incriminated inconsistent areas.^15^ It is therefore likely that not so much local damage, but rather a loss of network interactions contributes to patients’ symptomatology. Indeed, patients with TBI often present diffuse axonal injury (DAI), which produces a widespread structural disruption of connections between brain areas.^16^ This disruption impairs functional network interactions, measurable through functional connectivity (FC), defined as the temporal dependence of neuronal activity patterns across anatomically distinct brain areas.^17,18^ Previous studies have demonstrated that patients with moderate-to-severe TBI exhibit reduced FC compared with controls, in particular in the theta and alpha frequency bands.^19-21^ The theta and alpha frequency bands reflect cognitive and memory performance.^22^ However, these alterations have only rarely been linked to fatigue.

Recent evidence suggests that alterations of network interactions induce a pathological state of reduced brain excitability in the concerned patients.^20^ Patients with stroke show reduced excitability of the motor cortex, which correlated with a subjective experience of fatigue.^23^ In consequence, patients may have a reduced ability to induce efficient neural responses during task performance, in particular when they need to maintain effort. It is thought that patients can compensate for this on the short term by inducing particularly strong activations and interactions between brain regions at the beginning of a task,^24-26^ but this eventually leads to faster depletion of neural resources and appearance of fatigue.^27,28^

In sum, there is some evidence for neural alterations in patients with acquired brain lesions that might lead to inefficient task responses and, thus, reduced cognitive performance and fatigue. However, these hypotheses have so far only received fractional support. So far, no study has comprehensively assessed network and excitability states and neural activations over time while controlling for confounding covariates. As long as the neural origin of pathological fatigue is unknown, we will not be able to propose targeted interventions for these patients.

This study aimed to address this urgent need by investigating the neural correlates of subjective fatigue after TBI. To this end, we investigated three different populations: chronic TBI patients with subjective fatigue (TBI+), chronic TBI patients without subjective fatigue (TBI−) and age and gender-matched healthy controls (Healthy). Based on the abovementioned studies, we hypothesized that TBI+ patients have reduced FC between brain areas at rest^19-21^ and reduced cortical excitability^20,23^ compared with TBI− patients and Healthy. In addition, we expected increased activation at the beginning of a demanding cognitive task,^24-26^ followed by a faster depletion of neural resources as indexed by a loss of brain activation and FC towards the end of a cognitive task.^27,28^ FC and brain activation were reconstructed from EEG recordings at rest and during a two-back task, respectively. Excitability was quantified from the amplitude of motor evoked potentials (MEPs) induced by single-pulse transcranial magnetic stimulation (TMS) over the primary motor cortex and from resting threshold. We controlled for differences in cognitive performance, motor performance and subjective psychological covariates such as depression, anxiety and apathy. Cognitive performance was assessed with a two-back task during EEG as well as with comprehensive neuropsychological test battery including attention, executive and memory domains. Motor performance was quantified with Jamar dynamometer. Subjective psychological covariates were extracted from appropriate questionnaires. When the influences on fatigue following TBI are better understood, subsequent steps can be taken to address their modulation and treatment.

## Materials and methods

### Inclusion and exclusion criteria

The inclusion criteria were as follows: age 18–65 years, existing informed consent, chronic phase of TBI (>12 months after incident), previously having participated in rehabilitation because of the TBI, ability to participate and concentrate on a task for at least 60 min, Glasgow Outcome Scale Extended of five or more at the time the study sessions take place and normal or corrected-to-normal vision.

Exclusion criteria were as follows: reduced vigilance, inability to understand or follow procedures, concomitant progressive neurodegenerative brain disease, sleep disorders inducing excessive daytime sleepiness, regular consumption of benzodiazepines or neuroleptics, pacemaker or other active implants or metallic objects in the brain, skull breach such as craniotomy and pregnancy.

### Participants

Participants were recruited at the Division of Neurorehabilitation at the University Hospital of Geneva between April 2021 and June 2022. In total, 30 participants signed the informed consent. One control and one patient were excluded during the study due to non-participation in several assessments and EEG procedure. Ultimately, we included 17 chronic TBI patients and 11 Healthy. This convenience sample was part of a pilot study aimed at estimating the effect size for future, larger studies. The primary goal was to gather preliminary data to inform the planning and sample size calculations for adequately powered research. All participants were right-handed. Patients were divided into two subgroups based on the general fatigue score of the multidimensional fatigue inventory (MFI) questionnaire: TBI+ 12 or more points, TBI− <12 points.^29^ There were 10 TBI+ patients and seven TBI− patients. The three groups were comparable in terms of sex, age and education years but showed a statistically significant difference in the working capacity (see Table 1). Additionally, the time since accident and the distribution of brain lesions did not differ within the TBI groups. None of the patients took neuroleptics or sedatives. Two patients in the TBI+ group took antidepressive medication (serotonin reuptake inhibitors).

**Table 1:** Demographics and characteristics of the participants

	TBI+	TBI−	Healthy	Comparison between groups
*n*	10	7	11	
Sex				
Women	3	1	3	*χ* ^2^ = 0.6, *P* = 0.74
Men	7	6	8	
Mean age	47.4 ± 12.5	40.6 ± 15.1	46.1 ± 14.8	*F*(2,25) = 0.5, *P* = 0.61
Mean time since accident in months	56 ± 35	49 ± 15		*χ* ^2^ = 0.04, *P* = 0.85
Brain lesions				
DAI	7	6		*P* = 0.60
SDH	4	2		*P* = 1
SAH	5	5		*P* = 0.62
ICH	6	3		*P* = 0.64
Left	3	1		*P* = 0.60
Right	1	3		*P* = 0.25
Bilateral	6	3		*P* = 0.64
Medication				
Antidepressants	2	0		*P* = 0.49
Mean education years	15.2 ± 4.1	15.4 ± 3.3	14.4 ± 3.1	*χ* ^2^ = 0.6, *P* = 0.76
Working ability in %	**22 ± 33.6**	88.6 ± 22.7	69.1 ± 45.1	*χ* ^2^ = 9.6, *P* = 0.0083**

Bold values indicate significant differences (*P* < 0.05). **P < 0.01.

DAI, diffuse axonal injury; ICH, intracerebral haemorrhage; SAH, subarachnoid haematoma; SDH, subdural haematoma,

### Study protocol

**Figure 1:**
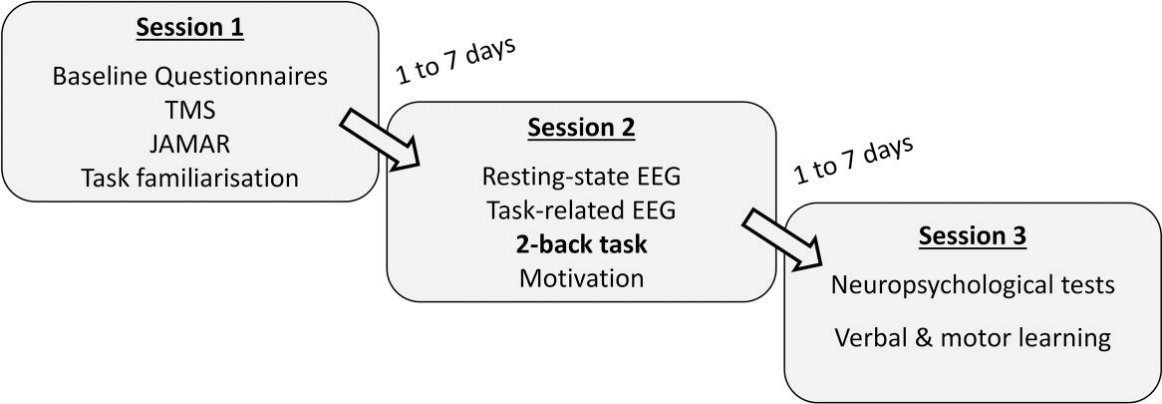
**Study protocol.** Session 1 contained psychological questionnaires, testing of motor excitability with TMS and the grip strength (Jamar hand dynamometer). In Session 2, EEG was performed before and during the two-back task. Session 3 contained neuropsychological tests. TMS, transcranial magnetic stimulation.

The participants performed three study sessions separated each by 1–7 days in the period from May 2021 to June 2022 (Fig. 1). During the first session, they had to fill out questionnaires on their psychological state (Table 2). Motor performance was measured at the beginning and the end of the first session as the maximum grip strength using a Jamar hand dynamometer. The excitability of the motor cortex was evaluated with single-pulse TMS. At the end of Session 1, the two-back task was explained to the participants, followed by a familiarization run. During the second session, task-related EEG was recorded while participants performed the two-back task. Resting-state EEG was obtained while participants were resting eyes closed before the two-back task, during a task break and after the task. At the end of Session 2, they filled the post-experimental intrinsic motivation inventory (IMI). Finally, during the third session, participants participated in several neuropsychological tests for their cognitive and learning abilities. Psychometrics of the questionnaires and assessments have been published elsewhere (see Tables 2 and  3).

**Table 2:** Psychological questionnaires

Questionnaire	Meaning	Comparison between the three groups
MFI total^30^	Subjective fatigue	*F*(2,25) = 24.0, *P* < 0.0001***
General fatigue		*F*(2,25) = 24.0, *P* < 0.0001***
Physical fatigue		*χ* ^2^ = 12.1, *P* = 0.002**
Reduced activity		*F*(2,25) = 3.8, *P* = 0.036*
Reduced motivation		*F*(2,25) = 8.9, *P* = 0.001**
Mental fatigue		*F*(2,25) = 10.3, *P* = 0.001***
FSS^5^	Subjective fatigue	*F*(2,25) = 22.5, *P* < 0.0001***
Epworth Sleepiness Scale^31^	Daytime sleepiness	*F*(2,25) = 0.7, *P* = 0.49
SMI^32^	Self-initiation and motivation	*χ* ^2^ = 4.1, *P* = 0.13
f-DAS total^33^	Subjective apathy	*F*(2,25) = 2.6, *P* = 0.10
Executive		*F*(2,25) = 4.0, *P* = 0.031*
Emotional		*χ* ^2^ = 0.3, *P* = 0.87
Cognitive		*F*(2,25) = 1.0, *P* = 0.38
HADS total^34^	Subjective anxiety and depression	*F*(2,25) = 4.7, *P* = 0.019*
Depression		*F*(2,25) = 4.1, *P* = 0.028*
Anxiety		*F*(2,25) = 2.8, *P* = 0.08
FrSBE total^35^	Subjective executive disorders	*F*(2,25) = 5.1, *P* = 0.014*
Apathy		*F*(2,25) = 13.4, *P* = 0.0001***
Disinhibition		*χ* ^2^ = 2.1, *P* = 0.36
Dysexecutive		*F*(2,25) = 6.4, *P* = 0.006**
24-item MacNair Scale^36^	Subjective memory impairment	*χ* ^2^ = 13.9, *P* = 0.001***
IMI-post total^37^	Motivation after performing the two-back task	*χ* ^2^ = 2.5, *P* = 0.28
Perceived competence		*F*(2,25) = 2.2, *P* = 0.14
Efforts engaged		*F*(2,25) = 2.3, *P* = 0.12
Pressure/stress felt		*F*(2,25) = 3.7, *P* = 0.039*
Likert scale change after–before two-back task	Subjective fatigue/motivation during the two-back task	
Fatigue		*χ* ^2^ = 4.2, *P* = 0.12
Motivation		*χ* ^2^ = 2.7, *P* = 0.26
PCA component 1	Subjective apathy, depression and anxiety	*F*(2,25) = 8.3, *P* = 0.002**

ANOVA or Kruskal–Wallis tests were used to compare between three groups (TBI+, TBI− and Healthy). Bold values indicate significant differences (*P* < 0.05). *P < 0.05, **P < 0.01, ***P < 0.001.

f-DAS, French Dimensional Apathy Scale; FrSBe, Frontal System Behavior Scale; FSS, Fatigue Severity Scale; HADS, Hospital Anxiety and Depression Scale; IMI, intrinsic motivation inventory; MFI, multidimensional fatigue inventory; PCA, principal component analysis of all non-fatigue questionnaires; SMI, Self-Motivation Inventory.

**Table 3:** Behavioural assessments for evaluating cognitive functions and motor performance

Behavioural assessment	Meaning	Comparison between the three groups
MNND^38^	Verbal episodic memory	
15 words, delayed recall		*χ* ^2^ = 5.2, *P* = 0.07
Grefex battery^39^	Auto activation and language production	
Phonemic verbal fluency		*F*(2,23) = 0.2, *P* = 0.8
Semantic verbal fluency		*F*(2,23) = 0.7, *P* = 0.49
Five Point Test^40^	Auto-activation	
Figural fluency productivity		*F*(2,23) = 1.2, *P* = 0.32
Colour Trails^41,42^ interference	Sustained and divided attention	*F*(2,23) = 0.1, *P* = 0.92
Stroop Victoria^43^	Selective attention and inhibition	*F*(2,23) = 0.6, *P*= 0.57
Digit symbol substitution task^44^	Processing speed, working memory, attention	*F*(2,23) = 1.9, *P* = 0.18
TAP alert without cue, median RT^45^	Intrinsic alertness	*χ* ^2^ = 6.5, *P* = 0.038*
TAP alert with cue, median RT^45^	Phasic arousal, temporal orientation of attentional focus	*χ* ^2^ = 3.6, *P* = 0.17
TAP divided attention, median RT^45^	Divided attention	
Audio stimuli		*χ* ^2^ = 1.7, *P* = 0.42
Visual stimuli		*χ* ^2^ = 6.5, *P* = 0.039*
TAP sustained attention, 3rd run, n° of errors^45^	Sustained attention	*χ* ^2^ = 0.01, *P* = 0.99
Two-back task^46^	Working memory	
Mean accuracy		*χ* ^2^ = 3.7, *P* = 0.16
TOT slope		*χ* ^2^ = 3.9, *P* = 0.14
FTT^47^	Motor learning	
Learning RT slope		*χ* ^2^ = 0.5, *P* = 0.79
Jamar dynamometer	Grip strength	*F*(2,25) = 0.9, *P* = 0.42

ANOVA or Kruskal–Wallis tests were used to compare between three groups (TBI+, TBI− and Healthy). Bold values indicate significant differences (*P* < 0.05). *P < 0.05.

DSST, digit symbol substitution task; FTT, finger tapping task; MNND, Materialien und Normwerte für die Neuropsychologische Diagnostik; RT, reaction time; TAP, test battery for attentional performance; TOT, time-on-task.

### Psychological questionnaires

Participants were asked to fill in questionnaires regarding medical and sleep history, fatigue [Fatigue Severity Scale (FSS) and MFI], day-time drowsiness (Epworth Sleepiness Scale), apathy [French Dimensional Apathy Scale (f-DAS)], self-initiative [Self-Motivation Inventory (SMI)], anxiety and depression [Hospital Anxiety and Depression Scale (HADS)], executive functions [Frontal System Behavior Scale (FrSBe)] and memory functions (24-item MacNair Scale) (Table 2).

### Behavioural assessments

#### Two-back task

**Figure 2:**
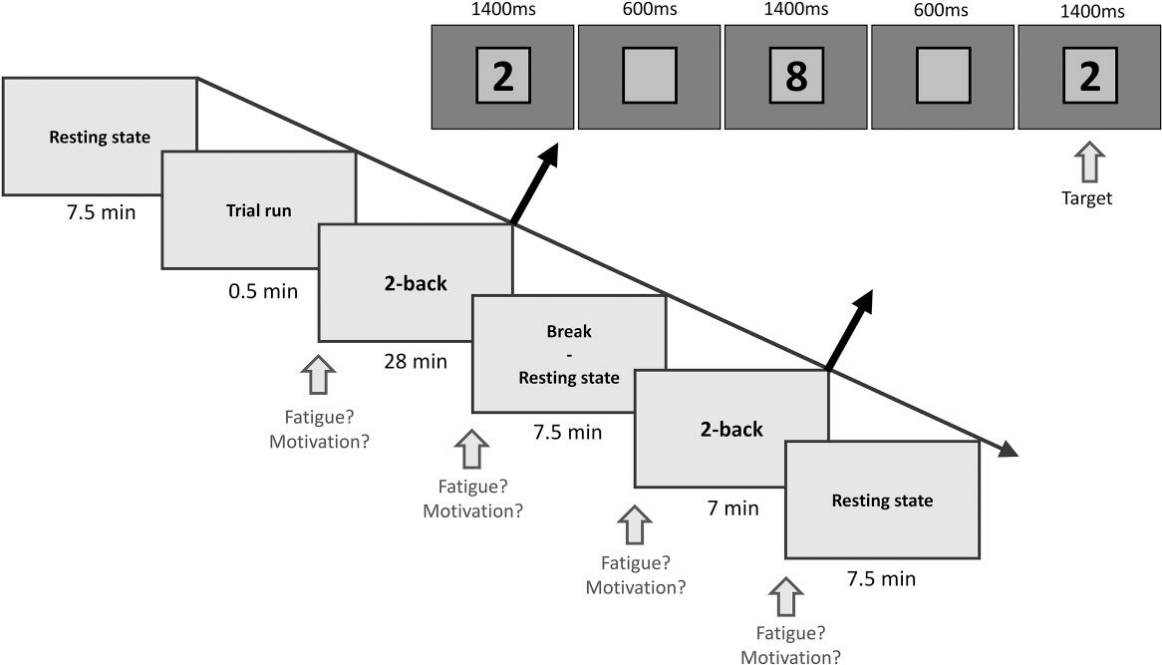
**Procedure of the two-back task during Session 2.** Participants performed the two-back task for 28 min (Part 1) and again for 7 min (Part 2) after a 7.5 min break. Participants had to press with their finger as fast as possible as soon as the previous to last number was identical to the current number. At the beginning and end of Parts 1 and 2, participants answered on a 1–5 scale how tired they were and how motivated they were to do the task (Likert scale).

Participants performed a two-back task aimed at delivering an objective measure of cognitive fatigue. This task imposes high and sustained attentional demands on working memory and attention, which are often impaired in TBI patients.^48,49^ In addition to the familiarization in session one, participants performed a 30 s trial run in the second session before starting the actual two-back task. Participants performed the two-back task for 28 min (part one), and again for 7 min (part two) after a 7.5 min break (Fig. 2). The task consisted of black numbers (1–9) appearing in font 150pt on the centre of a grey screen for 1400 ms with interstimulus interval of 600 ms. The participants sat 1 m away from the computer. There were 840 stimuli in Part 1 and 210 stimuli in Part 2. Participants had to press with their right index finger as fast as possible as soon as the previous to last number was identical to the current number. There were 140 targets in Part 1 and 35 in Part 2. During distractor numbers, participants had to refrain from pressing the button. To keep participants motivated, they received continuous feedback of their performance in the form of a coloured squared surrounding the number. Green, orange or red squares indicated good, medium or bad performance, respectively. Colour was adapted according to their accuracy over the past 20 stimuli and weighted on their response time. Threshold between green, orange and red feedback was adjusted on their mean response time determined in Session 1. The task was designed using E-Prime 2.0 software (Psychology Software Tools, Pittsburgh, PA, USA) and participants answered on a Chronos box (Psychology Software Tools, Pittsburgh, PA, USA; https://pstnet.com/products/chronos/).

#### Neuropsychological tests

Neuropsychological testing was obtained to evaluate the cognitive profile. The tests assessed verbal fluency (Grefex), 5-point figural fluency [Materialien und Normwerte für die Neuropsychologische Diagnostik (MNND)], inhibition (Stroop Victoria), attentional speed (Colour-Tray I and II), selective attention/working memory (digit symbol substitution task), alert [test battery for attentional performance (TAP)], divided and sustained attention (TAP) and motor learning [finger tapping task (FTT)] (Table 3). During the FTT, participants were instructed to repeat a given five-item sequence with their left, non-dominant hand (little finger to index) on four horizontally arranged buttons on a computer keyboard. They had to repeat the sequence as fast and accurately as possible during 10 blocks of 30 s separated by 30 s of rest. The sequence (1-4-2-3-1) was continuously presented to participants and did not change throughout the whole experiment. No feedback was given. Procedural learning was quantified as the reduction in reaction time from beginning to end, excluding errors. One TBI+ and one healthy participant refused the neuropsychological testing.

#### Grip strength

The maximal grip strength was measured with a Jamar hand dynamometer. The participants were seated with the arm in adduction, the elbow flexed at 90°, the forearm in the neutral position and the wrist between 0° and 30° of extension. To ensure reproducible measures, we repeated the measurement at two different time points separated by 2 h and obtained three measurements with each hand at each time point. The goal was not to find asymmetries between hands. Although eight TBI patients initially presented with mild hemiparesis, the asymmetry showed complete recovery in all. We thus averaged the sides to represent global bilateral grip strength.

### Neurophysiological assessments

#### Motor evoked potentials

The excitability of the motor cortex was quantified with a single-pulse TMS using a MagPro X100 stimulator (MagVenture A/S, Farum, Denmark) with biphasic waveforms and a MCF-B65 coil. MEPs were measured from the right first dorsal interosseous muscle for each arm. The surface electromyogram electrodes were placed in correspondence of the first dorsal interosseous muscle (active electrode) and over the associated joint or tendon (reference electrode), whereas the ground electrode was placed on the dorsal part of the forearm. The motor hot spot at the scalp location was searched and recorded by slightly moving the coil over the primary motor cortex area until MEPs of the greatest amplitude were found. To keep the location of the coil stable over the primary motor cortex, a neural navigation system (TMS Navigator, Localite, Bonn, Germany) was used. The location of the greatest amplitude was mapped on a template head, which was registered to anatomical landmarks on each participant. This method allows coil placement with reasonable accuracy. Then, the resting motor threshold (RMT) was defined as the lowest pulse strength at that site that produced MEPs with peak-to-peak amplitudes above 50 μV on at least six out of 10 trials and expressed as % maximum stimulator output. RMT was used as an indicator of cortical excitability. Finally, 20 MEPs were recorded at 100% RMT, 110% RMT and 120% RMT, and the average peak-to-peak amplitude across all stimulation intensities was extracted.

#### EEG

A resting-state EEG of 7.5 min duration with eyes closed was recorded before the two-back task, during the task break and after the two-back task, using a 128 electrode Active-Two system (Biosemi V.O.F., Amsterdam, the Netherlands) and a sampling rate of 1024 Hz. Additionally, EEG during the cognitive task was recorded. The EEG recording from one Healthy participant had to be excluded because of excessive noise.

### EEG analyses

Artefacts, including those caused by eye movements, blinks, power line interference, electrode noise and muscular activity, were removed through visual inspection for resting-state recordings and via independent component analysis (using the FastICA algorithm)^50^ for data recorded during the two-back task. Components were rejected based on their temporal characteristics and scalp topography. Persistently noisy electrodes were excluded from the analysis and data were re-referenced to Cz.

Source imaging was performed in MATLAB (The MathWorks), using the NUTMEG toolbox^51^ along with its Functional Connectivity Mapping module.^52^ Lead-potential calculations were performed with a boundary element head model, constructed using the Helsinki BEM library^53^ and the NUTEEG plugin of NUTMEG. The head model was derived from the standard Montreal Neurological Institute brain template. EEG signals were projected onto grey matter voxels using a scalar minimum variance beamformer,^54^ an adaptive filter that derives its weights from the sensor covariance matrix of the bandpass filtered data across all epochs.

For resting-state data, an ellipsoid filter was applied to bandpass the signals between 1 and 20 Hz. The filtered data were then segmented into 300 non-overlapping epochs of 1 s each. FC was quantified as the absolute imaginary component of coherency (IC),^52,55^ which reflects the stability of the phase differences over time. IC excludes zero-phase-lag coherence,^56^ thereby mitigating biases arising from volume conduction or spatial leakage of the inverse solution.^57^ Global FC at each voxel was expressed as weighted node degree (WND), calculated as the sum of IC values between the voxel and all other cortical voxels.^55,58,59^ WND provides an overall index of a voxel's role in brain network communication,^60^ abstracting from interactions with specific regions and accommodating individual variability in functional network topology. To control for potential variability in signal-to-noise ratio, WND values were normalized by *z*-scoring: each voxel's WND was standardized to the mean and standard deviation of all voxels of the subject's brain.^52,58^ The primary analysis focused on the alpha frequency band (8–13 Hz), which has been consistently linked to behavioural outcomes in both healthy individuals and patients.^55,61,62^ Alpha oscillations represent the dominant frequency for resting-state activity and are critical for functional interactions in the brain.^63^

Event-related power changes during the two-back task were evaluated to explore oscillatory modulations of sets of neurons indicating a local activation during the two-back task. Recordings during the task from two TBI+ patients had to be excluded because of excessive noise. Epochs were created from 300 ms before to 1400 ms after onset of the target or distractor. We bandpass filtered epochs in alpha (8–13 Hz) and beta (13–30 Hz) frequency bands, as a power decrease in these bands is indicative of local activation.^64^ Power was computed as root mean square values of the reconstructed source signals using a 300 ms long sliding window with 100 ms time steps. Values from both bands were averaged to obtain a single index of activation. The prestimulus baseline power was subtracted from all windows. Values were then averaged across epochs, separately for targets and distractors of the two-back task to assess differences in brain activation between targets and distractors. In addition, we also averaged across the combined targets and distractors to assess overall brain activation. To obtain a global index of brain activation, we averaged power change values across time windows between 0 and 1000 ms after stimulus onset. In an additional analysis to estimate time-on-task effects in power changes during the two-back task, we fitted a least-square regression across power change values in the successive epochs. Beginning-of-task power change was then given by the intercept, the change across time by its slope and end-of-task power change by the sum of intercept and slope.

### Statistical analyses

Values from all psychological questionnaire scores (excluding the fatigue questionnaires) were rank transformed and entered into a principal component analysis (PCA) using the singular value decomposition algorithm (pca() function of MATLAB). This yielded a first component explaining 42% of variance with high loadings for scores for depression (HADS depression), anxiety (HADS anxiety), apathy (f-DAS total and executive, FrSBE apathy), subjective executive disorders (FrSBE) and subjective memory disorders (MacNair). Scores in this first component were used as index for subjective covariates of fatigue.

Resting-state FC maps before the two-back task were tested voxel-wise for differences between TBI+ and Healthy with statistical non-parametric mapping. A correction for testing multiple voxels was obtained by defining a cluster-size threshold based on the cluster size distribution obtained after 5000 random permutations of original data.^65^ We then defined anatomical regions of interest (ROIs) approximating these significant clusters from an atlas.^66^ Average normalized WND values of the voxels belonging to the ROI were extracted for all participants and for all three resting-state recordings (before and after the two-back task as well as during the break after 28 min) and compared between groups.

To identify brain areas that activate during the two-back task in Healthy, we tested event-related power maps from combined target and distractor epochs for changes from baseline using statistical non-parametric mapping. A correction for testing multiple voxels was obtained by defining a cluster-size threshold based on the cluster size distribution obtained after 5000 random permutations of original data.^65^ Voxels belonging to the significant cluster were then defined as ROI and the mean power change of voxels in the ROI was extracted for all participants. Power values were log transformed to approximate normal distribution.

We then used Rstudio (version R 4.3.2) to test all variables from the psychological questionnaires as well as the behavioural and neurophysiological assessments for differences between groups. Normality was checked with the Shapiro–Wilk test. If the normal distribution was verified and no outliers were present, a one-way ANOVA between all three groups was performed. If the data were not normally distributed or in case of outliers, the Kruskal–Wallis test was used. Pairwise *post hoc* comparisons were performed with the Tukey–Kramer honestly significant difference test. Missing data were handled using a listwise deletion approach, in which cases with missing values were excluded from the respective analyses.

To identify independent predictors of fatigue, we included variables with significant between-group differences from the univariate analysis in a multivariate linear regression model. Fatigue was assessed using the total score of the MFI (MFI total) as the dependent variable. Neurophysiological predictors included alpha-band resting-state FC measured before the two-back task and the neural activation index during the two-back task across all participants. FC change from pre-task to the break was excluded due to its strong correlation with pre-task FC, ensuring the inclusion of only the most independent and predictive FC measure to avoid redundancy. Significant psychological covariates were combined into a single predictor using the first principal component derived from PCA. Behavioural performance variables (reaction times from the TAP alertness test without cue and the TAP divided attention test for visual stimuli) were averaged after normalization to *z*-scores to form a unified behavioural predictor. To account for potential differences between TBI patients and Healthy, we added a binary group membership variable (TBI versus. Healthy) as an additional factor in the model.

To check for specific associations with the individual fatigue subscales of the MFI as well as with the FSS, we additionally subjected significant predictors in the multivariate model to a Spearman rank correlation with all subscales, including values from all three groups.

## Results

### Psychological questionnaires

As expected, a strong between-group difference was observed in most fatigue subscales of the MFI (see Table 2 and Supplementary Fig. 1). It is however noteworthy that no difference was found in the ‘reduced activity’ subscale. No significant difference between groups was detected in daytime sleepiness (Epworth Sleepiness Scale). The executive functions were perceived as impaired by TBI+ patients in two different questionnaires (f-DAS and FrSBE), particularly in terms of apathy and planning and initiation of problem-solving strategies. The MacNair Scale indicated strong differences in subjective memory function (*P* = 0.0009). The general motivation to complete a task showed no significant difference (SMI, IMI-post), but TBI+ patients differed significantly in the perceived competence of finishing the task. TBI+ individuals felt significantly more depressed than TBI− or the Healthy, and there was a non-significant trend towards more anxiety (HADS). We were able to combine these subjective impairments in a single PCA component, which has high loadings in depression, anxiety and apathy. As expected, the PCA scores showed also a significant difference between groups.

### Behavioural assessments

The neuropsychological assessments revealed a significant difference between groups regarding intrinsic alertness (TAP alert without cues, *P* = 0.039) and divided attention for visual stimuli (TAP visual stimuli, *P* = 0.039). There were no significant group differences in all other neuropsychological assessments nor in the motor evaluation regarding the grip strength (see Table 3 and Supplementary Fig. 2). The two-back task did not reveal differences in mean accuracy or performance degradation over time (time-on-task) between groups.

### Neurophysiological assessments

Motor excitability assessed with the MEPs showed no significant difference in the group comparison (see Table 4).

**Table 4:** Neurophysiological assessments

Neurophysiological assessment	Meaning	Comparison between the three groups
MEP	Motor excitability	
Threshold mean right/left hand		*F*(2,23) = 0.6, *P* = 0.55
Peak-to-peak amplitude		*χ* ^2^ = 0.1, *P* = 0.97
Alpha-band FC pre	Resting-state network interaction before the two-back task	*F*(2,24) = 6.4, *P* = 0.006**
Alpha-band FC change	Change in resting-state network interaction from before to after the two-back task	*F*(2,24) = 6.1, *P* = 0.007**
Mean alpha/beta power change	Neural activation during the two-back task	*χ* ^2^ = 6.9, *P* = 0.032*

ANOVA or Kruskal–Wallis tests were used to compare between three groups (TBI+, TBI− and Healthy). Bold values indicate significant differences (*P* < 0.05). *P < 0.05, **P < 0.01.

MEP, motor evoked potentials; FC, functional connectivity.

**Figure 3:**
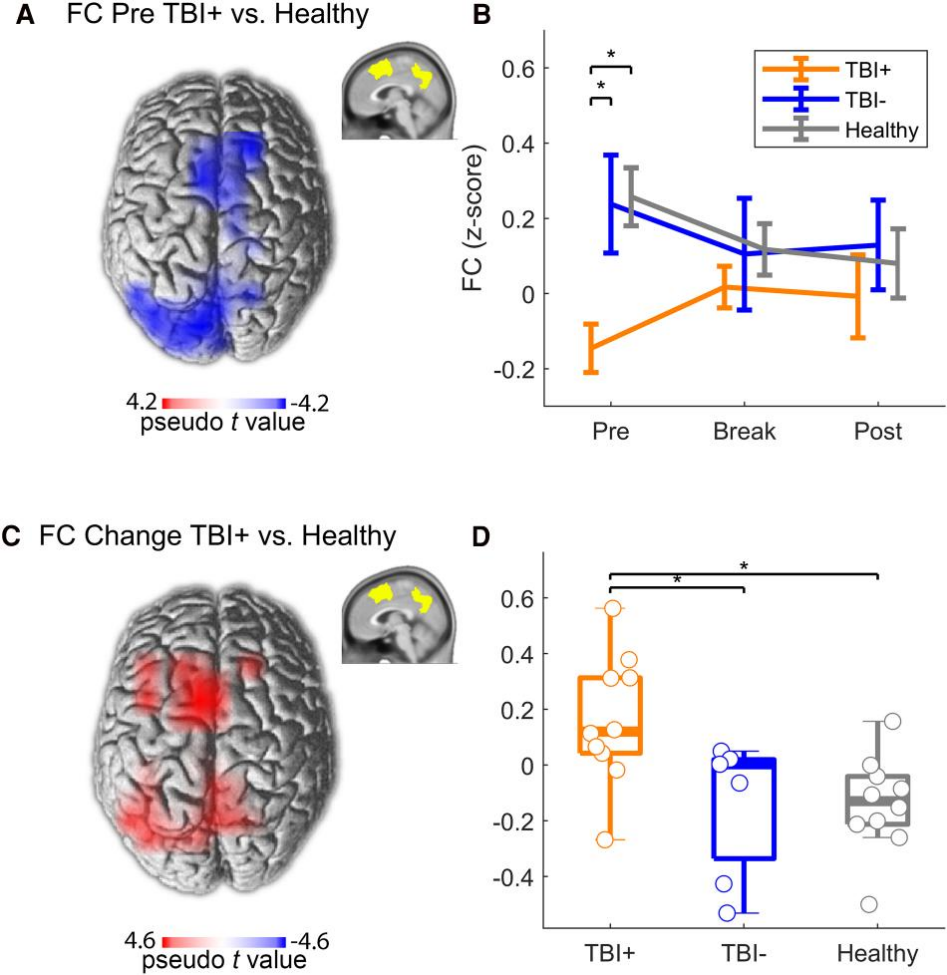
**EEG network correlates of fatigue after TBI.** (**A**) Difference in global alpha-band FC between TBI+ (*N* = 10) and Healthy (*N* = 10) during rest before the two-back task (statistical non-parametric mapping, *P* < 0.05, cluster-corrected). Supramodal medial cortical areas (inlay) were defined as ROI using anatomical templates. (**B**) Alpha-band FC (mean ± standard error) between the ROI and the rest of the brain before (pre) and after (post) the two-back task, as well as during a break after 28 min for TBI+ (*N* = 10), TBI− patients (*N* = 7) and Healthy (*N* = 10). * indicate significant differences in Tukey–Kramer honestly significant difference pairwise comparisons (*P* < 0.05). (**C**) Difference in FC change from before the two-back task to the break between TBI+ (*N* = 10) and controls (*N* = 10). (**D**) Change in alpha-band FC between the ROI and the rest of the brain for the three patient groups. The asterisks (*) indicate significant differences in Tukey–Kramer honestly significant difference pairwise comparisons (*P* < 0.05). Boxes indicate the median, 25th and 75th percentile of the data. Circles represent values from individual participants. FC, functional connectivity; TBI, traumatic brain injury.

A significant disruption in global alpha-band FC in bilateral medial parietal, prefrontal and premotor areas was found in TBI+ compared with TBI− and Healthy before the two-back task in the resting-state EEG (Fig. 3A; Table 4, ‘alpha-band FC pre’).

However, TBI+ patients increased resting-state alpha-band FC of these areas after performing the two-back task (Fig. 3B, ‘break and post’), such that the difference between groups disappeared during these recordings. Thus, TBI+ patients increased their FC significantly more from before the task to the break than TBI− and Healthy in the same brain areas (Fig. 3C and D; Table 4, ‘alpha-band FC change’).

**Figure 4:**
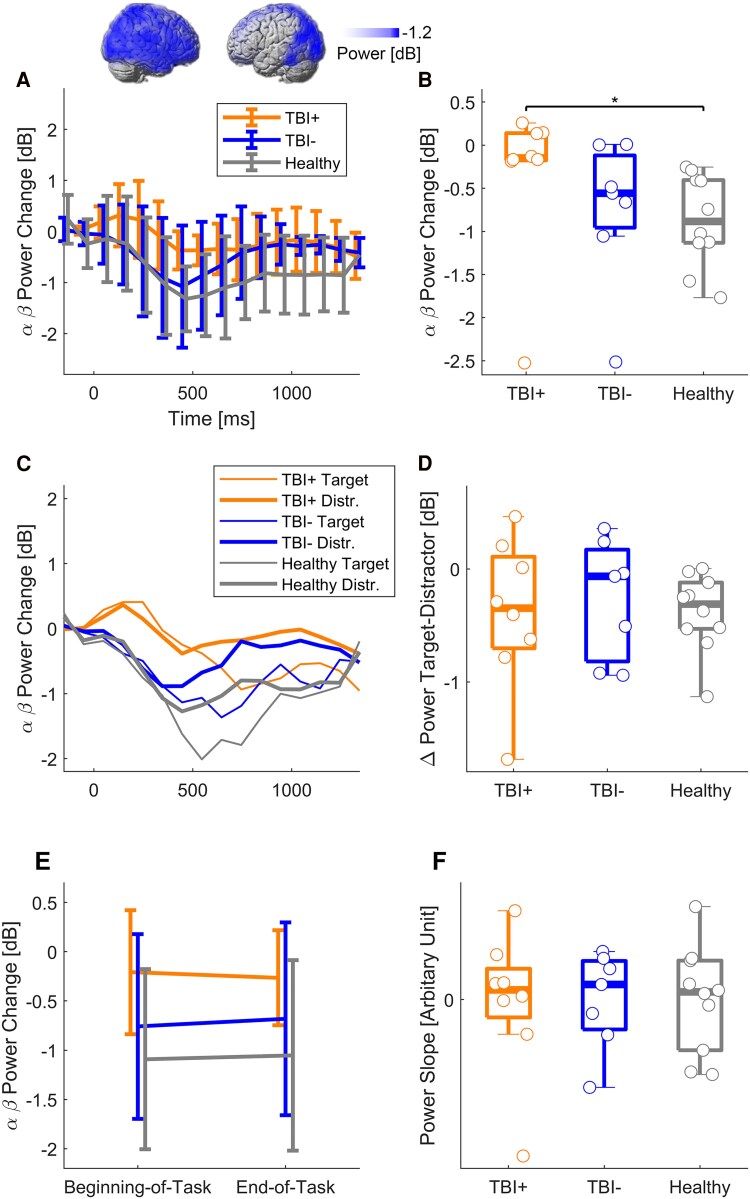
**Brain activation during the two-back task in TBI patients and healthy controls.** (**A**, *top*) The two-back task induced widespread neural activation (coloured areas) as measured with alpha and beta power decrease in the right hemisphere and the posterior left hemisphere in Healthy (statistical non-parametric mapping, *N* = 10, *P* < 0.05, cluster corrected). (**A**, *bottom*) Activation peaked 500 ms after onset of the target or distractor in the three participant groups. Error bars show median ± interquartile range. (**B**) TBI+ patients (*N* = 8) exhibited reduced signs of overall brain activation compared with Healthy (N = 10). * indicates significant difference in the Tukey–Kramer honestly significant difference test (*P* < 0.05). Boxes indicate the median, 25th and 75th percentile of the data. Circles represent individual subjects. (**C**) Targets were associated with greater activation in those widespread brain areas than distractors in all participant groups. (**D**) The power difference between targets and distractors did not differ between participant groups (Kruskal–Wallis, *P* = 0.85). (**E**) Task-induced power decrease remained stable in all participant groups but was globally smaller in TBI + . (**F**) There was no group difference in the slope of power change from the beginning of the task to the end of the task (Kruskal–Wallis, *P* = 0.97). TBI, traumatic brain injury.

Brain activation, as indexed by alpha/beta power decrease during the task, differed significantly between groups (Fig. 4A). Individuals with TBI+ exhibited reduced signs of overall brain activation compared with Healthy (*χ*^2^ = 6.9, *P* = 0.032) (Fig. 4B). Across all three groups, the brain was more strongly activated during a target than a distractor (*P* = 0.005) (Fig. 4C), but this did not differ between groups (*χ*^2^ = 0.3, *P* = 0.85) (Fig. 4D). There was no difference in the slope of power change in the group comparison from the beginning to the end of the two-back task (*χ*^2^ = 0.1, *P* = 0.97) (Fig. 4F).

### Multivariate regression

Finally, we created a multivariate model of neural correlates of subjective fatigue that included psychological and behavioural covariates. As hypothesized, we found that resting-state FC before the two-back task was a significant independent predictor of fatigue as measured with the MFI. Moreover, the PCA component with loadings for depression, anxiety, apathy and subjective executive disorders was also significant (Table 5). The adjusted *R*^2^ value (0.80) indicates a high explanatory factor of the predictor variables in the multivariate regression model.

**Table 5:** Multivariate regression

Multivariate regression	Estimate	SD	*t*-value	*P*-value
Mean TAP	1.14	1.54	0.74	0.47
Alpha-band FC pre	−13.88	5.04	**−2.75**	0.013*
Mean alpha/beta power change	4.39	2.24	1.97	0.07
PCA component 1	0.39	0.07	**5.34**	< 0.0001***

Predictors with bold values are significant (*P* < 0.05). *P < 0.05, ***P < 0.001.

Multivariate regression with the dependent variable MFI total and the predictor variables mean TAP (mean of the z-transformed value TAP without cue median RT and TAP divided attention visual stimuli), alpha-band resting-state FC before the two-back task, mean alpha/beta power change and PCA component 1.

PCA, principal component analysis; TAP, test battery for attentional performance.

To check for a possible moderating influence of including TBI patients and Healthy in the multivariate regression model, we additionally added the dichotomous factor patient versus healthy to the model. In this case, the diagnosis factor was not significant (*P* = 0.82) and did not interact with any of the other factors (*P* > 0.45). Furthermore, the same predictors remained significant: ‘alpha-band FC pre’, *P* = 0.029; and ‘PCA component 1’, *P* < 0.0001. The diagnosis TBI therefore did not influence the dependent variable fatigue by itself. Additionally, the adjusted *R*^2^ value declined to 0.79.

### Correlations with sub-dimensions of fatigue

**Figure 5:**
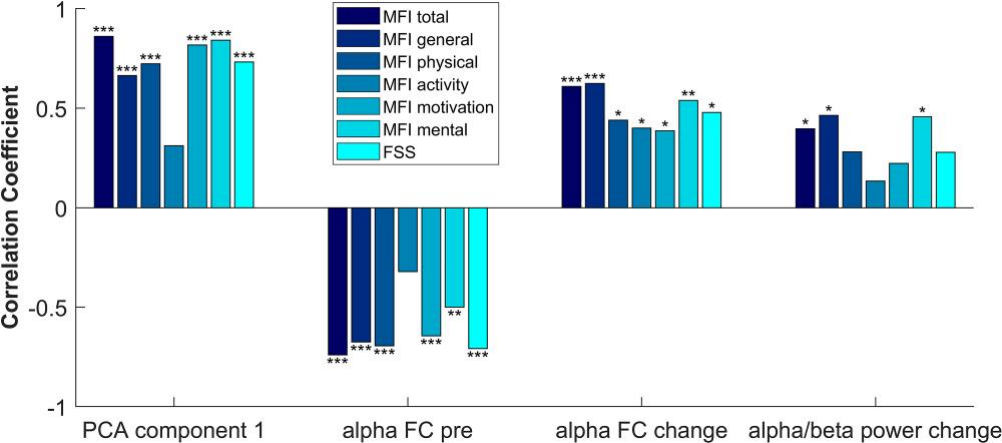
**Bivariate Spearman's rank correlation between predictors and different subscales of fatigue.**  *N* = 28 (PCA), *N* = 27 (alpha FC pre and alpha FC change) and *N* = 25 (mean alpha/beta power change). Level of significance (*P*-value): *<0.05, **<0.01 and ***<0.001. FC, functional connectivity; FSS, Fatigue Severity Scale; MFI, multidimensional fatigue inventory; PCA, principal component analysis of all non-fatigue questionnaires.

To investigate differences across various dimensions of fatigue, we additionally computed bivariate correlations. Results reveal that psychological covariates and neural predictors correlate with most subscores of the MFI as well as the FSS (Fig. 5). The subscore ‘reduced activity’ shows no significant correlations with the predictors except for ‘alpha FC change’. The variable ‘mean alpha/beta power change’, which stands for the neural activation during the two-back task, shows only borderline significant correlations with MFI general and MFI mental fatigue.

## Discussion

The aim of this study was to investigate the contribution of neural alterations to the subjective experience of fatigue after TBI to pave the way towards new treatment targets. Previous studies had suggested multiple factors, neural as well as behavioural and psychological that may be associated with fatigue. Here, we aimed at bringing the pieces together by using a multivariate framework and by investigating the link between different measures of neural processing, behaviour and subjective experience in TBI and neurotypical adults.

The main neural predictor of fatigue that came out in the multivariate regression was a disruption of global resting-state alpha-band FC (‘alpha-band FC pre’ task) between supramodal medial cortical areas and the rest of the brain in TBI+ patients compared with the other groups (Fig. 3A and B). Cortical midline structures are known to be principally involved in self-related processing (e.g. hearing one's own name and seeing one's own face).^67^ They are also part of the so-called default-mode network. Qin and Northoff^67^ suggested that our sense of self may result from interactions between resting-state activity of the default-mode network and stimulus-induced activity within the midline regions. Shan *et al*.^68^ showed that default-mode network activity is more complex and less coordinated in patient with diagnosed chronic fatigue syndrome, indicating brain network analysis could be potentially used as a diagnostic biomarker. We can therefore speculate that network perturbations of cortical midline structures could reflect altered inferences of patient's own level of fatigue. Studies further suggest a central role of the precuneus in highly integrated tasks, including visuospatial imagery, episodic memory retrieval and self-processing operations.^69^ Thus, the observed neural alterations may also be linked to complex cognitive disturbances. Another midline region is the supplementary motor area. The supplementary motor area has a rich network of white matter connections with motor, language and limbic areas.^70^ The area plays an important role in planning, initiation and execution of movements and speech. The supplementary motor area through its connections to the cingulate gyrus may also play a role in motor processing of negative emotional stimuli.^71^ Hinds *et al*.^72^ suggested that vigilance disturbances are related to functional changes in the default-mode network and the supplementary motor area. Thus, the altered resting-state alpha-band FC observed here may influence action planning, cognitive processing, emotional regulation and the representation of the self.

We can speculate that the disruption of neural interactions may in turn result from DAIs, which are indeed frequently observed in TBI^16^ or from loss of grey matter volume.^73^ In addition to the initial DAI, Johnson *et al*.^74^ found ongoing white matter degeneration for many years after TBI. Green *et al*.^73^ suggested that this secondary degradation could be due to reduced physical and cognitively demanding activity after TBI, which may offset neuronal proliferation and survival. Xia *et al*.^75^ described abnormal FC density in DAI patients, which could be related to impairment of consciousness and cognition. It is important to emphasize that the abnormal FC reported here was observed only in TBI+ patients, not in TBI patients in general, thus suggesting a specific impact on fatigue.

TBI+ patients were able to compensate the spontaneous reduction of neural interactions, as suggested by the increase of FC between the same brain regions and the rest of the brain immediately after a task (Fig. 3B, ‘alpha-band FC change’). The compensatory increase in FC agrees with findings of previous studies. Ramage *et al*.^28^ reported increased FC in a cingulo-opercular network during a cognitive task in mild TBI patients compared with neurotypicals when analysing functional MRI (fMRI). Taken together, this suggests that upon task demands, TBI patients can upregulate neural interactions, but this may be associated with effort and thus fatigue. Indeed, we observed a significant correlation between the increase in neural interactions and subjective fatigue (Fig. 5). It is however noteworthy that the Likert scale of fatigue and motivation, which the participants answered before and after the two-back task, showed no group difference. The TBI+ patients were therefore not more fatigued by or less motivated to do the task itself. The questionnaire measuring subjective motivation (IMI) also did not show a group difference in total, but the TBI+ felt significantly less competent after the two-back task (Table 2 and Supplementary Fig. 1). Hence, the subjective differences do not concern fatigue states after tasks, but trait fatigue as measured with the MFI and FSS questionnaires. This points to alterations in longer-term self-related representations, which fit well with the alterations in cortical-midline structures observed here.

We also observed altered neural activations during a demanding cognitive task in TBI+ patients. Individuals with TBI+ exhibited reduced signs of widespread brain activation compared with neurotypicals in the univariate group comparison (Fig. 4, ‘mean alpha/beta power change’). The reduced activation was observable not only during targets but also during distractors without need for action. This suggests a global reduction of neural activation independent of the task demand or behavioural actions. Previous studies have also reported reduced EEG indices of brain activation in TBI patients.^76^ Conversely, previous fMRI studies rather showed increased brain activation in moderate-to-severe TBI patients during a cognitive task.^26,77^ The increased fMRI activation was found to be unrelated to task accuracy and therefore interpreted as an inefficient utilization of neural resources. It is currently unclear how we can reconcile the opposite observations in EEG and fMRI studies.

It is further noteworthy that our EEG markers of reduced brain activation did not show time-on-task effects; i.e. they were present equally at the beginning and at the end of the task, and there was no difference in the slope of neural activation over time. Therefore, the hypothesis of faster depletion of neural resources during a cognitive task cannot be confirmed in our sample with our EEG measures. Neural depletion has previously been described in healthy subjects performing prolonged tasks with high load as decreased fMRI activation in the left inferior frontal gyrus, striatum and the cerebellum^27^ or as decreased theta oscillations.^78^ Some studies have questioned the reproducibility of neural depletion in neurotypicals.^79^ We are not aware of any study demonstrating neural depletion (reduced neural activation over time) in TBI. On the contrary, Olsen *et al*.^80^ reported greater activation increase over task duration in TBI than neurotypical in right frontal and parietal brain areas.

Our results on neural activation during the two-back task have to be interpreted with caution, as no behavioural difference in reaction time or errors between patients and controls was observed. This may suggest that it was not long/demanding enough for inducing fatigue in our sample. It is possible that longer or more demanding expositions to tasks would be needed in TBI before neural depletion can be observed.

Unlike previous studies,^20,23^ we did not observe reduced excitability of the motor system as assessed with MEPs in TBI+ patients (Table 4). Previous studies had a larger patient sample, and our study may have been underpowered to detect such differences. Our patient sample may also have been less severely affected. However, this suggests that motor excitability as assessed with MEPs is less strong predictor than neural interactions obtained from EEG.

TBI+ patients were remarkably similar to the other groups in the objective cognitive assessments. We found few differences in the neuropsychological tests. None of the assessments survived as significant predictor in the multivariate regression model. This is in agreement with Dillon *et al*.,^11^ who describe a relatively weak evidence for a relationship between cognitive impairment like information processing, attention, memory and executive function and higher fatigue levels. Thus, it seems that fatigued patients can compensate for neural alterations to reach comparable performance as controls. One of the underlying mechanisms may be the upregulated neural interactions, which we observed in TBI+ patients (Fig. 3B–D).

Significant neuropsychological dysfunctions were found in tests of intrinsic alertness and divided attention (Table 3 and Supplementary Fig. 2), while executive functions showed no significant difference between groups. Previous studies reported attentional-executive impairment in stroke patients with fatigue,^14^ while fatigue after TBI was related to processing speed, working memory and attention.^81^

Depression, anxiety, apathy and subjective executive and memory disorders co-varied with fatigue in our sample, in agreement with numerous previous studies. There has been a long-standing debate on the causality of this correlation with some studies suggesting that fatigue may be the cause rather than the consequence of depression and anxiety.^8-10^ Our study suggests that both fatigue and other psychological complaints may all be linked to a common neural disturbance. We observe that the disruption of neural interactions of supramodal cortical midline structures predicts fatigue independently of the psychological covariates. In addition, it correlates also with the scores of the first principal component representing depression, anxiety and apathy (*rho* = −0.59, *P* = 0.001). This may suggest that disrupted neural interactions are a common underlying correlate of fatigue as well as other psychological complaints. It is not possible from neuroimaging findings to draw conclusions on causality. The disturbances may be the consequence of encoded reductions in endurance that were experienced over prolonged periods by the patients. Alternatively, FC disruptions may cause disturbed self-related processing as discussed above. In any case, the findings suggest that treatments should attempt to work on self-related cognition. In addition, they may target neural interactions directly, for instance with neuromodulation techniques. Neuromodulation like repetitive TMS, transcranial direct current stimulation, transcutaneous vagus nerve stimulation and neurofeedback was found to be effective regarding pain and headaches, dizziness, depression, anxiety, sleep disturbance, general disability, some aspects of cognition, return to work and quality of life in mild TBI patients.^82^ Porcaro *et al*.^83^ suggested personalized neuromodulation may be an effective treatment for fatigue with multiple sclerosis. Non-invasive neuromodulation can also influence brain network interactions.^84-86^

The small sample size constitutes the main limitation of the current study. Although our results suggest interesting links between neural function and fatigue, a greater sample size will be necessary to demonstrate the generalizability and external validity of the study results. Since all patients were selected from one rehabilitation unit in the same hospital, selection bias could occur. Furthermore, the cognitive testing done in this study does not adequately capture a full day at work and might be underdosed to induce fatigue and lacking performance. One could also consider other covariates of fatigue such as for example oculomotor function, inflammation, stress levels and others.

Our findings should encourage larger, adequately powered multicentre studies, ideally incorporating improved methods for assessing objective cognitive fatigue, such as prolonged task exposure or increased cognitive demand. Reliable tests for objectively measuring cognitive fatigue are still lacking, highlighting the need for improved assessment methods.

In conclusion, our findings suggest that the disruption of global resting-state alpha-band FC is a key neural predictor of fatigue post-TBI. The patients can upregulate and compensate this altered neural state by the increase of FC during a cognitive task. Conversely, neural activation during a task and cognitive performance show less associations with fatigue after TBI. The network disturbances in the medial cortical areas could influence self-related processes, episodic memory, planning, initiation and execution of movements, speech and emotional regulation. If these findings can be confirmed in larger samples, new treatment options for fatigue post-TBI could try to correct the observed network disturbances. Treatment approaches of fatigue after TBI could contain psychological programmes working on altered representation of the self or propose techniques such as non-invasive neuromodulation to influence the network interactions.

## Supplementary Material

fcaf082_Supplementary_Data

## Data Availability

Data will be made available upon request. EEG analyses were made with the open-source toolbox NUTMEG (https://www.nitrc.org/projects/nutmeg/). The code used in Rstudio for the multivariate regression can be found in the Supplementary material.
