# Supplementary material for: Neural correlates of fatigue after traumatic brain injury
Source: Brain Commun. 2025 Feb 26;7(2):fcaf082. doi: 10.1093/braincomms/fcaf082 (PMC11891517; doi:10.1093/braincomms/fcaf082)
Supplement: fcaf082_Supplementary_Data [file fcaf082_supplementary_data.docx]

**Supplementary material to**

**Neural correlates of fatigue after traumatic brain injury**

*Annina E. Anliker,^1,†^ Léa A. S. Chauvigné,^2,†^ Leslie Allaman,^2^ and Adrian G. Guggisberg,^2,3,*^*

**Code**

The code used in Rstudio for the multivariate regression was:

lm(`MFI total`~ `mean_z_TAP`+ `alpha FC pre` + `mean alpha/beta power change`+ `PCA component 1`, data = data4)

**Supplementary Figures**

**
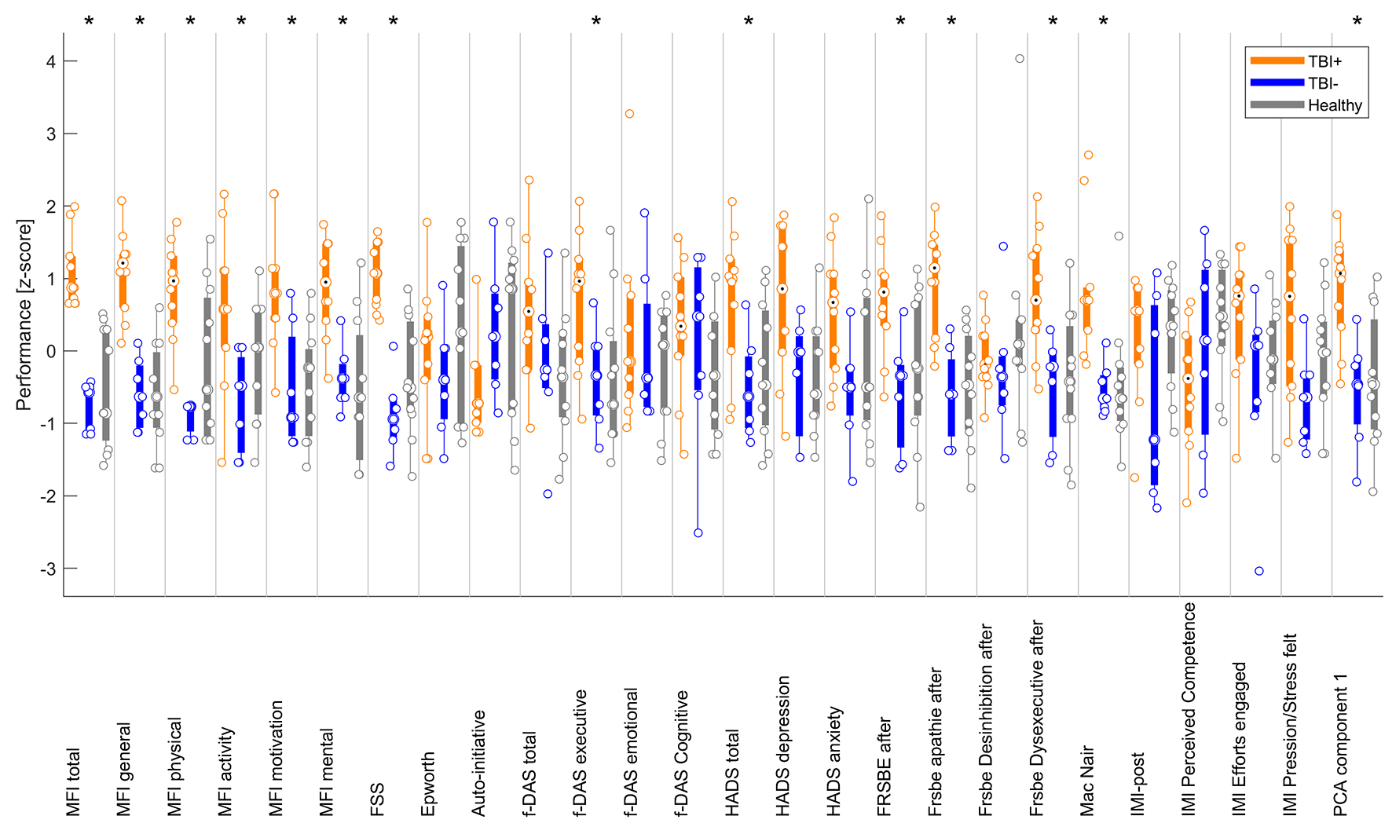
**

**Supplementary Figure 1: distribution of the subjective impairment in psychological questionnaires across groups.**

* significant difference between TBI+ (N=10), TBI- (N=7) & Healthy (N=11), p<0.05 (Kruskal Wallis test). Circles indicate data points from individual participants.

*Abbreviations*: MFI, multidimensional fatigue inventory; FSS, fatigue severity scale; f-DAS, French dimensional apathy scale; HADS, hospital anxiety and depression scale; Frsbe, Frontal System Behavior scale; IMI, intrinsic motivation inventory; PCA, principal component analysis of all non-fatigue questionnaires.


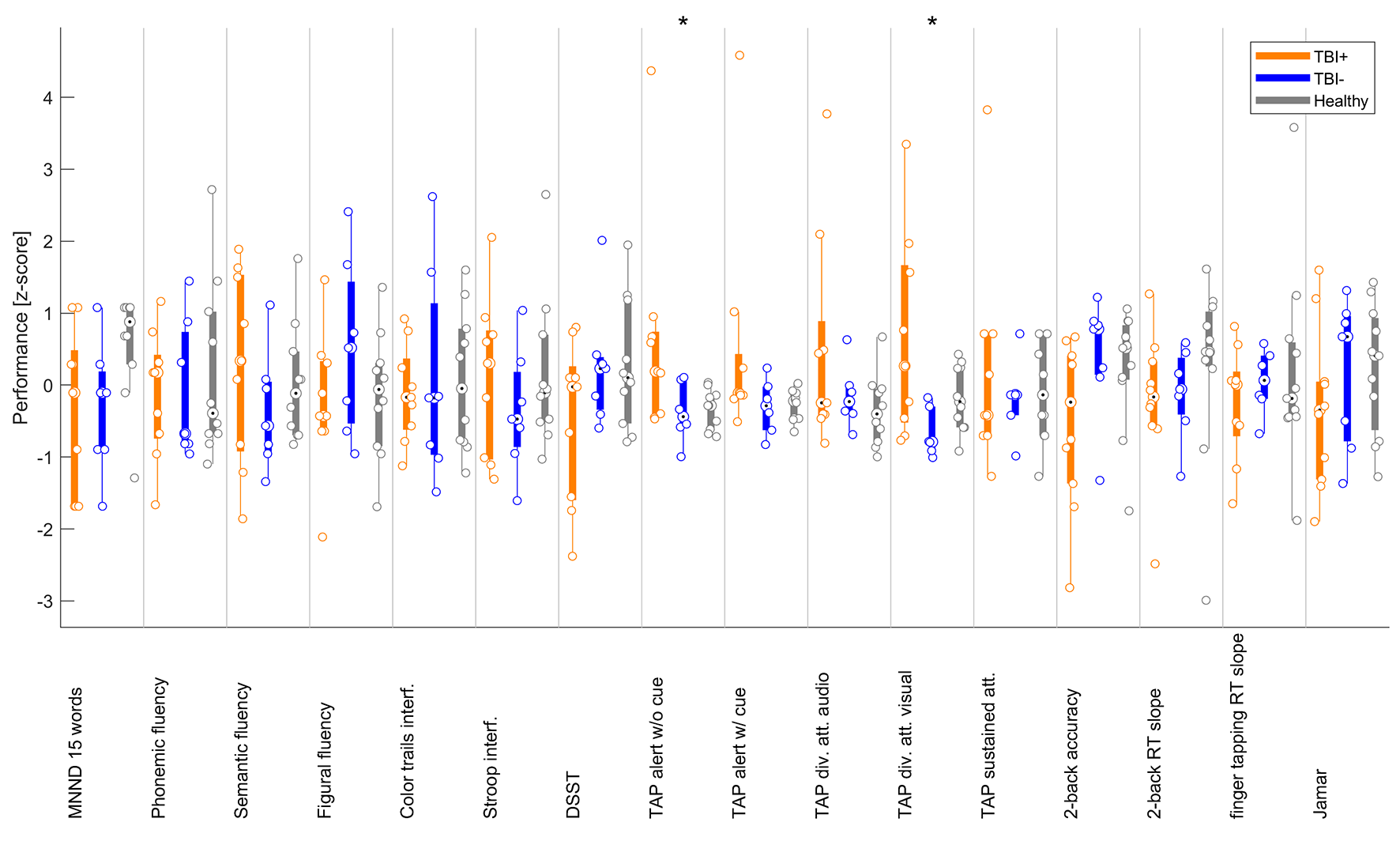


**Supplementary Figure 2: Distribution of performance in the behavioral assessments across groups.**

* significant difference between TBI+ (N=9), TBI- (N=7) & Healthy (N=10), p<0.05, Kruskal Wallis test. Circles indicate data points from individual participants.

*Abbreviations*: MNND, Materialien und Normwerte für die neuropsychologische Diagnostik; DSST, digit symbol substitution task; TAP, test battery for attentional performance; RT, reaction time.
